# An overview of Indian research in schizophrenia

**DOI:** 10.4103/0019-5545.69229

**Published:** 2010-01

**Authors:** Parmanand Kulhara, Ruchita Shah, K. R. Aarya

**Affiliations:** Department of Psychiatry, Postgraduate Institute of Medical Education and Research, Chandigarh - 160 012, India

**Keywords:** Schizophrenia, India, Research

## Abstract

The Indian Journal of Psychiatry published three articles in its first issue way back in 1958. Since then, it has steadily published more than 200 papers on one or the other aspect of schizophrenia. From rudimentary research methodology and descriptive approach, schizophrenia research, as published in the Journal, seems to have come of age with more and more sophisticated research designs and methodologies. Our ardent researchers have made significant contributions in the understanding of this riddle called schizophrenia. Notable contributions have been made in the field of epidemiology, course and outcomes and phenomenology of this disorder. However, research in psycho-social rehabilitation of schizophrenia and related areas is sparse and sporadic. The need to conduct research that impacts health policies and planning of services for this disorder is evident and our researchers would do well to provide impetus in these areas.

## INTRODUCTION

The Indian Journal of Psychiatry has come a long way from its humble beginning with the publication of its first volume in October 1958. The inaugural issue had three articles on schizophrenia, the first, an Editorial Annotation titled “Tranquillizers” by Dr. Kirpal Singh, which drew attention to the phenomenon of “market being flooding with numerous so called tranquillizers,” though Dr. Kirpal Singh observed even at that time that the efficacy of these and relative superiority of any one of these were questionable.[1] He pondered over whether continued search for an “ideal” tranquillizer would yield fruitful results and whether it will be a boon or a curse! In the second article, Prof. S.M. Bose, Professor of Psychotherapy at Medical School, University of Zurich, Switzerland wrote on the topic of the role of psychotherapy in schizophrenia in which the author described his experience of psychoanalytically oriented psychotherapy in a case of schizophrenia as a case illustration and claimed achieving very good result with this approach.[2] The third article was by A.K. Lalkaka, in which he described a case of schizophrenic reaction with peculiar visual disturbances.[3]

Annotation of research in various aspects of schizophrenia published in the Indian Journal of Psychiatry, since its inception, follows the above mentioned prelude. We have adopted a descriptive approach, describing the research published in our Journal and offer comments on the research efforts where appropriate.

### The 1960s

The articles published in the Journal in the 1960s were an assortment of publications related to the etiology, clinical aspects, treatment and, course and outcome of schizophrenia.

### Etiology

#### Era of psychodynamics

Evident from the writings of the sixth and seventh decades of the last century, psychodynamics and psychotherapy had important places in the field of schizophrenia. However, no significant research endeavors were made to study the dynamics of schizophrenia in a systematic manner. Rao[4] the only one to have published in this area, employed the PF-16 questionnaire in schizophrenic patients and found that the schizophrenic personality was characterized by low ego strength and low intelligence.

### Cultural factors

Teja[5] showed that the first and second born sibs had greater risk of developing schizophrenia, in contrast to the finding of western studies and hypothesized that this possibly reflected cultural expectations and the way in which the child is brought up.

### Moving towards neurobiology

Biological experiments conducted elsewhere started finding mention in the abstracts by eminent Indian psychiatrists. Kirpal Singh[6] provided an abstract of Nichol C’s work where the author had concluded that there was some material present in the urine of these patients that inhibited normal glucose utilization by mouse brain extract. He also provided another similar abstract dealing with plasma protein fraction.

### Clinical considerations

A paper by Deb[7] gave apt description of clinical features of schizophrenia at a time when operationalized criteria for the diagnosis of this disorder were yet to materialize. The author recognized that the ‘prepsychotic personality’ is of ‘introvert’ type and scholastic decline invariably occurred with frequent changes of job.

Narayana Murthy[8] studied communication in schizophrenia and found that most disturbances in communication were in the form of inhibition of speech. This study had a large sample and intelligence was also assessed and taken into consideration.

### Treatment

Clinical observations rather than ‘trials’ were the main basis for guiding management of patients with schizophrenia. Catatonia was thought to be aggravated by Rauwolfia and/or Largactil.[7]

An eight-week placebo controlled study of prochlorperazine carried out by Menon[9] in 20 females with schizophrenia showed that 80% patients had variable response.[9] Another trial[10] in 30 chronic treatment resistant schizophrenic patients and five treatment resistant manic patients showed that thioproperazine was valuable in treating both sets of patients but had severe extra-pyramidal effects.[10] Both the studies had small samples and no control groups. Another study[11] also used quantitative measure of improvement.[11] Towards the end of this decade of 1960s, comparative trials also surfaced, for example the drug trial of prochlorperazine versus chlorpromazine in a randomized fashion[12] Narayana Murthy[13] (1966) found no significant impairment in intellectual functioning or memory following ECT.

### Course and outcome

Dutta Ray and Kapur[14] conducted a study to delineate prognostic factors in schizophrenia and this work made important contributions to the understanding of this complex illness. It was stressed that the chances of improvement were significantly higher in patients with shorter duration of illness (less than one year), no family history of mental illness, acute onset and younger age whereas gender and previous history of psychotic illness had no prognostic significance. The study had several methodological strengths; a large sample size, standard and uniform treatment (a combination of up to 10 ECTs and up to 300 mg/day of Chlorpromazine, no formal psychotherapy in any case), a prospective study design with a follow-up of six months.

### Comments

The research publications of the 60s are creditable for various reasons. Even though research tools and funding were not available and though the research mostly followed a descriptive paradigm, it is praiseworthy that people even then had enthusiasm and enterprise. These publications are now of historical value contributing richly to the history of psychiatric research in our country.

### 1970s

#### Epidemiology

Prabhu and Ramchandran[15] in a sample of 125 patients, found that there was over-representation of first-borns in contradiction to western studies thus raising questions regarding socio-cultural differences.

### Biological research

Cytogenetic scientists conducted research on dermatoglyphics in schizophrenia and found that the patients had higher indices as compared to normal controls.[16] Eswaraiah[17] (1978) in a Council of Scientific and Industrial Research funded study found that patients had higher frequency of single radial base crease than controls. As in the last decade, biological research continued, and a study[18] was conducted to compare ABO blood group and secretor/non-secretor status of schizophrenics with normal controls and found secretor status was more common in O positive schizophrenics. In another study, Sethi *et al*.[19] found lymphocytic abnormalities in patients and their family members; pointing towards genetic factors in the causation of schizophrenia. Prakash and Sethi[20] studied pattern of serum proteins in schizophrenia by means of a controlled design; Verghese and Thomas[21] studied histamine tolerance, while Sridhar Rama Rao *et al*.[22] (1976) studied 3, 4-dimethoxy phenyl-ethylamine excretion in these patients.

A family study detecting psychiatric morbidities in family members of schizophrenia patients was carried out in a sample of 500 patients by Sethi *et al*.[23] Though commendable, the main drawback of this study was that only 22.8% of the relatives were directly interviewed; while for the rest, data were gathered from informants, thus, reducing credibility of the research findings.

### Phenomenology

Bhaskaran and Saxena[24] compared male and female schizophrenia patients and showed that symptoms at the onset of illness did not differ with gender, but, catatonic schizophrenia occurred more frequently in females and precipitating factors were commoner in female patients. Another such attempt was made by Sharma and Gupta[25] who found that different types of delusions occurred in different types of families. This added to the clinical observation that content of delusions is affected by socio-cultural factors.

Akhtar *et al*.[26] attempted to explore the relation between obsession neurosis and schizophrenia and found that none of the 44 patients with obsession symptoms developed schizophrenic symptoms two to nine years after initial contact.

Singh *et al*.[27] constructed a battery of five psychometric tests in Hindi and carried out quantitative and qualitative psychometric assessment of disorders of form of thought in 30 schizophrenia patients and compared with normal controls. The analysis revealed perseveration, circumstantialities and irrelevancy in schizophrenics.

Varma *et al*.[28] studied concretization of thinking and found low abstract ability in schizophrenics as compared to normal controls and neurotics. Besides, the interest in phenomenology was reflected by detailed clinical descriptions of 13 cases of pseudoneurotic schizophrenia by Varma *et al*.[29] Abraham *et al*.[30] used Repertory Grid Technique for the assessment of schizophrenic thinking; but the sample size was small, while Chatterjee and Thakur[31] studied the Muller-Lyer illusion in 40 subjects.

### Treatment

Therapeutic efficacy of thiothixene was compared to five phenothiazenes in 60 chronic schizophrenia patients by Kishore *et al*.[32] Prochlorperazine was found to be the most efficacious. This study used randomized sample and standardized instrument (Psychotic Symptom Rating Scale) to measure improvement; but, the sample size was too small (10 patients in each group) for any meaningful statistical comparisons. Other studies with pimozide (Kishore and Dhillon,[33] Mahal and Janakiramaiah,[34] used Brief Psychiatric Rating Scale and special mental status questionnaire, and Wing’s Rating Scale. Ramachandran and Sarada Menon,[35] Sharma and Dutta,[36] Bagdia *et al*.,[37] conducted controlled drug trials; however, the main methodological limitations in most of these studies were small sample size and short period of follow-up and assessment. Bagadia *et al*.[38] conducted a trial on Flupenthixol and considered it particularly useful in drive deficit schizophrenia.

Clinical trials of depot preparations had begun in the 70s. A trial of fluphenazine enanthate of four-week duration with a follow-up of eight weeks was carried out by Bagadia *et al*.[39] involving 50 patients. Overall improvement was noted in 78% of the patients; however, the follow-up period was short. Another drug trial was conducted by Jha and Bhaskaran[40] in 112 long stay patients and yet another such trial was carried out by Bagadia *et al*.,[41] but with a small sample size of 27 schizophrenia patients.

Controlled trial[42] of multiple stimuli ECT showed that it was more effective than standard single stimulus ECT.

### Course and outcome

In a, longitudinal study (involving 128 patients with a follow-up period of 1½ to 2½ years aimed at delineating good prognostic factors, Narayan *et al*.[43] (1974) tested factors proposed by Robin and Guze. About 2/3^rd^ of the patients were followed up and outcome was defined a *priori*; a positive relation was found between good prognostic factors and outcome. Sarada Menon and Ramachandran[44] compared social and clinical characteristics of 96 long stay schizophrenia patients and a group of 103 schizophrenia patients who were discharged within six months of admission and found that marked social withdrawal and socially embarrassing behavior, low work output, lack of social contact before and after admission and unemployed status at the time of admission affected the outcome and prolonged hospital stay.

### Miscellaneous

Rastogi and Mahal[45,46] studied relationships in 12 families with schizophrenia patients and compared with 12 families who had neurotic patients. Poor parental functioning, poor marital relations and skewed family dynamics were revealed in these studies reflecting the then prevalent hypothesis of dysfunctional family as a cause of schizophrenia. Another study[47] showed that parental deprivation was more common in the childhood of schizophrenia patients; however, a retrospective study design is a weakness of the study.

Mazumdar and Sinha[48] conducted a study in which various components of Rorschach test and dynamic factors were studied in paranoid schizophrenia patients.

Varma[49] studied the effect of antipsychotic on EEG changes in stage 1 sleep and found that the percentage of stage 1 sleep increased steadily; however, the analyzable sample had only six patients.

Bhaskaran *et al*.[50] studied the effects of prolonged institutionalization on 148 patients with schizophrenia and found that majority of the patients were indifferent to discharge and were almost totally neglected by the relatives.

### Comments

The research carried out in the 70s failed to adhere to a rigorous methodology, compounded by the fact that diagnosis of schizophrenia lacked reliability. Dermatoglyphics and phenomenology dominated research efforts. However, there was no unifying trend in the research carried or reported.

### 1980s

#### Epidemiology

Padmavathi *et al*.[51] (1988) carried out a large epidemiological study in urban Madras and reported that prevalence of schizophrenia was highest among functional psychoses, at 2.49/1000 population. A higher rate was seen in males and in the age range of 15-45 years. Door to door survey conducted on a large sample using standardized instruments, and stringent criteria for case identification and diagnoses are the strengths of the study.

### Biological

Various studies were reported in the Journal in search of biological markers and diagnostic tests in schizophrenia. Kondaiah *et al*.[52] found that plasma Creatine Phosphokinase (CPK) levels were higher in the patients suggesting a diagnostic value of CPK. Ghosh *et al*.[53] found that patients with schizophrenia excreted greater amount of Vanilmandelic Acid (VMA) and lower amounts of steroid fractions as compared to controls. Significantly lower levels of CSF 5-hydroxy indole acetic acid (5 HIAA) were found in patients as compared to controls by Pandey *et al*.[54] and Tiwari *et al*.[55] found increased levels of serum and cerebrospinal fluid immunoglobulins; whereas Narasimha Rao *et al*.[56] observed no significant differences in immunoglobulin levels between schizophrenia (paranoid and non-paranoid) patients and normal controls. Platelet monoamine oxidase activity was studied in chronic schizophrenia patients[57] and no differences were documented between the patients and normal subjects. Serum prolactin level was also studied in treatment naive and medicated patients[58] and no differences were found between un-medicated patients and control subjects, refuting the hypothesis that there is a generalized hyper dopaminergic state.[58] Chatterjee[59] studied dopamine related hormones- prolactin, growth hormone and luteinizing hormone in 84 patients with acute schizophrenia. Electroencephalogram patterns were studied by Gaur and Bhat[60] who found left hemispheric laterality in seven out of 35 drug-free patients.

The soft neurological signs (SNS) and minor physical anomalies (MPA) were studied in 107 adult schizophrenia patients by Nizamie *et al*.[61] SNS were found more commonly in chronic schizophrenics as compared to normal subjects; the most prevalent SNS being those related to sensory integration and motor co-ordination. Also, the mean MPA score was higher as compared to reported score in normal populations.

Sethi *et al*.[62] conducted family study assessing psychiatric morbidity among parents of patients with schizophrenia and reported higher incidence of psychiatric morbidity in the parents. Ponnudurai[63] studied 102 families of schizophrenic patients by drawing pedigree charts and concluded that the findings favored a polygenic inheritance. Nuclear sexing and karyotyping was done in 287 patients wherein patients with schizophrenia showed more prevalence of chromatin positive than controls.[64] Some genetically determined somatic traits such as ear lobes, finger hair and hairy ear were studied in schizophrenia.[65] The proportion of schizophrenic patients with ear lobes hanging free was much less than the controls. The presence of physical anomalies was investigated in 80 patients with schizophrenia and the incidence was found to be significantly high.[66] Jangid and Verma[67] compared 380 schizophrenic subjects with 685 controls with respect to season of birth and found an excess of winter births in the former group by 6.1%. Such studies advanced research in the biological understanding of schizophrenia.

Jayaswal *et al*.[68] studied structural changes in brain using computed tomography and found that the size of lateral ventricles expressed as ventricular brain ratio, and the width of the third ventricle and the sylvian fissure were significantly greater in schizophrenia patients.

### Phenomenology

Kulhara *et al*. studied the phenomenology of delusions in 112 patients with schizophrenia using the Present State Examination. The authors found that the delusions of persecution were more common in males and those above the age of 30 years. Educated patients had more delusions of reference, delusional misinterpretation and delusions of thoughts being read. Also, systematization was seen more in younger patients, while married patients had more delusions of reference. Kulhara *et al*. presented the usefulness of the Brief Psychiatric Rating Scale (BPRS) in positive and negative sub-typing of schizophrenia as significant differences emerged between the two sub-types on items like emotional withdrawal, guilt feelings, tension, hallucinatory behavior, motor retardation, blunted affect and excitement. Also, Kota and Kulhara[71] used a cross-sectional phenomenological approach in 40 schizophrenia patients and found that on variables like age, duration of illness, and premorbid adjustment, significant differences emerged between positive and negative subtypes. Psychiatric manifestations of 72 CATEGO Class S+ schizophrenia were studied by Kulhara *et al*.[72]. Significant cross-cultural differences were revealed as it was observed that Indian CATEGO S+ patients had significantly less depression, tension and anxiety. Differences in nuclear syndrome and Schneider’s First Rank Symptoms were also observed.

Mazumdar *et al*.[73] assessed thought, language and communication disorders in 45 schizophrenia patients and observed that positive and negative thought disorders were in equal proportion in both positive and negative schizophrenics. Poverty of speech, derailment, loss of goal and perseveration were found to be the commonest thought disorders.

Ramanathan[74] studied auditory hallucinations in 30 patients with schizophrenia and found that these were more real than unreal. Also, neuroticism scores[75] were positively related to the anxiety prior to the ‘voices’, anticipation of the voice and interference of activities of the patient by the voice. Singh and Sachdeva[76] after assessing clinical criteria, family history, treatment response and follow up criteria suggested that schizoaffective schizophrenia was closer to primary affective disorders rather than being considered as a subtype of schizophrenia. This study gives a glimpse of the probable future controversies regarding status of schizoaffective disorders and the affective-psychosis spectrum.

A study[77] comparing negative symptoms in schizophrenia and depression revealed that the global ratings for affective flattening, alogia, avolition and inattention were significantly higher in schizophrenics, whereas anhedonia-asociality was equally commonly seen in depression. Also, the awareness of negative symptoms was higher in depression. Raju[78] studied depression in 146 patients of schizophrenia by regular follow-up of six months or more and described that schizophrenia patients manifested depressive symptomatology at first presentation and over a course of time following treatment. Also, it was observed that ‘neuroleptics may have a role in the causation of depressive disorders in schizophrenia.

### Psychological

Kota *et al*.[79] carried out a study to test the inter-rater reliability of Scale for Assessment of Negative Symptoms and found that the inter-rater reliability for all the items was significantly high ranging from 0.96 to 0.59. In a study by Ramanathan[80], high scores on psychoticism as measured by Eysenck’s Personality questionnaire seemed to be associated with greater number of languages of the ‘voice’, known living ‘speakers’, less fear and passive listening during auditory hallucination and coping with auditory hallucinations was also studied.[81]

### Psychosocial

The child-rearing attitudes and personalities of 100 pairs of parents of patients with schizophrenia of either gender were compared with controls and significant differences were found on variables like irritability, suppression of anger, suppression of sex, avoidance of communication etc.[82,83] Suman *et al*.[84] studied social problems of patients and their families and discussed interventions to manage these problems.

Trivedi *et al*.[85] studied attitudes of caregivers towards the patients and observed that the frequencies of critical comments, hostility, and emotional over-involvement were higher though not statistically significant in ‘relapsed’ or ‘continuously ill’ patients.

Compared to families of patients with chronic lung disease, the families of the patients with schizophrenia felt greater burden in the areas of finance, family leisure, family interaction and mental health of other family members according to a study by Gautam and Nijhawan.[86] Mubarak Ali and Bhatti[87] found no relation between family burden experienced by the caregivers and social support system as perceived by the patients. Bhargava *et al*.[88] found no differences in the social network of schizophrenia and non-schizophrenia psychiatric patients.

### Treatment

As in the last decade, drug trials of various oral and depot preparations[89] of antipsychotics continued in the 1980s. A comparative study[90] showed superiority of chlorpromazine to lithium in terms of improvement in acute schizophrenia. Also, controlled, double blind trial[91] of penfluridol, a long acting oral antipsychotic, was carried out, while Sharma[92] evaluated this drug in 29 patients with schizophrenia.

A comparative study[93] showed no differences in the efficacy of spaced versus daily electroconvulsive treatment in patients with schizophrenia. Janakiramaiah and Subbakrishan[94] compared ECT- Chlorpromazine combination to Chlorpromazine alone and found an earlier onset of response in the ECT group but the differences in response became non-significant at six weeks. Similar findings were reported Natani *et al*.[95] with three-arm study-haloperidol alone, ECT alone and haloperidol-ECT combination in 90 patients. Agarwal and Winny[96] studied combined ECT- Chlorpromazine regimen with a follow-up period of one month; but did not find any advantage of ECT over chlorpromazine alone. Taking cognizance of side effects of various antipsychotic drugs, Kuruvilla[58] found increased serum prolactin levels in medicated patients, while Nizamie and Sharma[97] observed that there were ECG changes in patients on Trifluperazine.

### Course and outcome

Verghese *et al*.[98] reported a multicentric study that examined the factors affecting the course and outcome of schizophrenia and found better outcome in the patients compared to reported findings from the West. Thara *et al*.[99] studied relapses in schizophrenia using the life chart method and found a steep fall in the percentages of non-relapsers from 85% at the end of first year as compared to 35% at the end of third year.

Kulhara *et al*.[100] examined the relation between measures of outcome and socio-demographic and diagnostic variables in schizophrenics. The authors found that gender of the patient, place of origin, lack of persistence at work, poor premorbid work record, hospitalization at the time of intake, loss of interest, affective flattening and incoherent speech had prognostic implications.

### Rehabilitation

Nagaswami *et al*.[101] studied the rehabilitation needs of schizophrenia patients and found that employment and vocational needs were highest and women and men had identical needs. On assessing the performance of 129 schizophrenia patients, Gopinath *et al*.[102] found that poor social support and presence of residual symptoms were significantly related to poor performance.

### Comments

Biological and phenomenological investigations dominated research in the decade of the 80s. Biological research was mostly directed towards replication of the research findings of the West although it did not match research sophistication and the technical fastidiousness of the Western researchers. Nonetheless, our pioneering researchers in this area were able to generate research funding, thus, forcing research funding agencies to take a more benevolent attitude towards research in mental health. Phenomenological research carried out during this period on the other hand had more sophisticated research designs, trusted and tested research instruments and adherence to some or the other operationalized criteria for the diagnosis of schizophrenia.

Phenomenology research in this era brought comparative or transcultural psychiatry in sharp focus, compelling outsiders to take serious notice of similarities as well differences in phenomenology between the East and the West. Course and outcome studies provided the much needed independent support to “better outcome” hypothesis. Despite the publication of a few studies, research in psychological, psychosocial and rehabilitation aspects of schizophrenia continued to be neglected areas. Drug trials were few but had improved research designs and assessment though the numbers of patients studied in these trials were modest. ECT research had few dedicated workers and their research output is commendable.

### 1990s

#### Epidemiology

As part of the World Health Organization (WHO) collaborative study, two geographically defined populations in urban and rural Chandigarh (North India) were monitored for a period of two years. The annual incidence rates obtained were 4.4 and 3.8 per 10,000 for the rural and urban areas respectively.[103] As part of an ICMR funded longitudinal study in urban Madras (now Chennai), Rajkumar *et al*.[104] found an incidence rate of 2.1 per 10,000 by the community survey and 4.1 per 10,000 by the leakage study.

### Biological research

As in the previous decade, Jhingan and Munjal[105] studied dermatoglyphics in patients with catatonic schizophrenia, whereas Jain *et al*.[106] studied palmer flexion crease pattern in schizophrenia subjects, their relatives and normal controls. Studies in dermatoglyphics were also reported by Sengupta and Das Bhuyan[107] and Ponnudurai *et al*.[108] in familial and non-familial schizophrenia. On studying Smooth Pursuit Eye Movements (SPEM), Sharan *et al*.[109] found that 73.33% of the schizophrenia patients as compared to 40% of normal controls had ‘impaired’ pursuit performance. On similar lines, Shaji *et al*.[110] found significantly more neurological abnormalities in schizophrenia patients and their first degree relatives as compared to normal controls. Radio Isotopic Technique was employed to measure Platelet Monoamine Oxidase activity by Sharma *et al*.[111] and no differences were found between schizophrenic, manic and control subjects. However, the same group[112] also found that the enzyme activity was significantly lower in paranoid group as compared to the other groups. Also, significant negative correlations between enzyme activity and severity, and duration of illness were found. Pradhan *et al*.[113] studied Plasma Homovanillic acid levels in schizophreniform patients, schizophrenia patients ‘on medications’ and schizophrenia patients ‘off medication. Similarly, concentrations of Homovanillic acid and gonadal hormones were studied by Gong *et al*.[114] Agarwal *et al*.[115] conducted a double blind trial of an immunomodulator combined with chlorpromazine versus chlorpromazine-placebo combination and found that the change in T-suppressor cell counts correlated significantly with improvement in psychosis and proposed that the findings supported the autoimmune hypothesis.

Ananthnarayan *et al*.[116] studied clinically remitted outpatient schizophrenia patients on measures of visual information processing and found that they performed poorly when compared to neurotic depressives. Kurup *et al*.[117] studied serum digoxin levels in 25 patients to support the hypothesis that hypothalamic digoxin dysfunction occurs in schizophrenia. Borde *et al*.[118] used the happy-sad chimeric faces test to elicit left hemifacial bias (LHF bias) and found that manics and schizophrenics did not show significant LHF bias.

Neuroimaging had begun to be used as a research tool by this time. Siddhartha *et al*.[119] studied computed tomography in 50 patients with schizophrenia and found abnormalities as compared to control subjects. Lal *et al*.[120] studied neurological soft signs, cognitive dysfunction and ventricular brain ratio in schizophrenia patients.

Somanath *et al*.[121] conducted a comparative family study to determine independence or overlap of familial risk for schizophrenia and bipolar disorders.

Thus, research in India reflected the drive to find biological markers of schizophrenia in common with elsewhere in the world. Improvement in methodology; use of advanced techniques, comparatively larger sample size were also visible.

### Phenomenology

With revisions of classifications, more emphasis was laid on delusions and hallucinations for diagnosis of schizophrenia. Singh and Kulhara[122] presented four cases of ‘simple schizophrenia’ and argued in favor of retention of this category. As ‘acute and transient psychosis’ became a distinct category in ICD 10, Janakiramaiah *et al*.[123] carried out a study to compute the discriminant validity of acute psychosis diagnosed as schizophrenia according to ICD 9.

Borde *et al*.[124] reported that negative symptoms and syndromes were more stable over time as compared to positive symptoms, and both positive and negative subtypes were stable. Kulhara *et al*.[125] compared the clinical features of late-onset schizophrenia (LOS) and early onset schizophrenia (EOS) and found that LOS group had higher score only on ‘persecutory delusion’ which led them to conclude that the findings did not support the diagnostic validity of LOS. When subjects of schizophrenia and depression were compared[126], the frequency of suicide attempts was higher in depressed patients (7.2%) than schizophrenic patients (4.7%). Tharayan and Kuruvilla[127] studied the correlates of depressive syndrome in patients with schizophrenia and found that mild depressive states were more commonly associated with retained insight and lower negative symptom scores. On studying obsessive compulsive symptoms in schizophrenia, Jaydeokar *et al*.[128] found that in patients with more than five-year duration of illness, 26.7% had OC symptoms and further commented that OC symptoms were more prevalent among paranoid schizophrenia patients; obsessions of contamination, sexual and aggressive thoughts and compulsion of need to ask or confess being more common.

Giridhar *et al*.[129] found a higher linguistic competence in positive schizophrenics as compared to the other groups and also reported that high linguistic competence was an indicator of poor prognosis in positive schizophrenics and of a better prognosis in negative schizophrenics. Aga *et al*.[130] examined the relationship of insight with psychopathology in 59 patients and found that insight had significant positive associations with number of previous episodes and treatment taken in the past. The phenomenological presentation of late onset schizophrenia was studied in 59 patients by Harish *et al*.[131], who reported that the commonest phenomenon were persecutory delusion followed by delusion of influence and hallucination in any modality.

### Psychology

On Luria - Nebraska Neuropsychological Battery (LNNB), schizophrenia patients performed better than brain damaged patients but worse when compared to normal controls.[132] The findings of this study indicated high discriminative accuracy and clinical effectiveness of the LNNB in Indian population.[132]

### Course and outcome

In a five-year follow-up study, Verghese *et al*.[133] found that about 67% of the patients had good outcome and reported regular drug compliance, short duration of illness, absence of economic difficulties, absence of dangerous behavior and delusions of persecution at intake, acute onset, and younger age of onset predicted good outcome.

Thara and Rajkumar[134] studied the characteristics of the attrition sample while carrying out a prospective study to examine the course of schizophrenia and found that the clinical course determined the regularity. The same authors[135] measured disability on longitudinal follow-up and found that the three-year course of disability tended to be stable and that disability did not seem to be related to relapses. Also, Thara and Joseph[136] compared the course in males and females and found that males had more disability, especially in the area of occupational functioning.

### Psychosocial factors and rehabilitation

Among families of long-term psychiatric patients, Roychaudhuri *et al*.[137], found that the burden was higher among care-givers of patients with schizophrenia. Sam *et al*.[138] assessed rejection response in relatives of patients and found it to be related to gender of patients and relatives (higher in males). Chakrabarti *et al*.[139] compared the extent and pattern of family burden in affective disorders and schizophrenia and found that both the subjective and objective burden were more in relatives of schizophrenia, family routine, family interaction, family leisure and finances were the areas in which burden was primarily felt. Kulhara *et al*.[140] assessed life events and social support in married schizophrenia patients and reported higher stress score and greater number of undesirable life events in the married group.

Padmavati *et al*.[141] developed the SCARF Social Functioning Index to measure social functioning and assessed the reliability and validity of the instrument. Thara *et al*.[142] devised the Burden Assessment Schedule (BAS) to assess the subjective and objective burden on caregivers of mentally ill patients.

Thara and Srinivasan[143] studied marital rates and their correlates in a cohort followed up for 10 years; a high rate of marriage was found in patients and majority of the marriages were intact at follow up. The same authors[144] evaluated an intervention program comprising social skills training, family education, occupational training and medication management training.

### Treatment

Borde *et al*.[141] attempted to predict the outcome of schizophrenia by subjective response to a test dose of Haloperidol after four hours and found that a dysphoric response predicted a poor therapeutic outcome. Such studies, despite methodological limitations, exemplify the constant search for predictors of response to antipsychotics. Dua *et al*.[146] in a controlled study did not find any advantageous or deleterious effects of adding imipramine to chlorpromazine in the treatment of depressive symptoms of schizophrenia.

Towards the end of the decade, newer antipsychotics (second generation) had made entry. In small open trials of risperidone, the drug was found to be both efficacious and safe.[147–149] Singh *et al*.[150] in a double blind trial comparing centbutindole and haloperidol found these to be equally efficacious. Emmanuel *et al*.[151] studied effectiveness of loxapine in a six-week open trial in 66 patients with schizophrenia. Clozapine was tested by Desai *et al*.[152] in treatment resistant cases and 25-50% decline in the BPRS score was noted, and sedation and sialorrhea were seen to be the most common side-effects. An open clinical trial on clozapine was conducted by Agarwal *et al*.[153], in a group of treatment resistant patients,[153] while Srinivasan and Latha[154] reported a case of use of clozapine in childhood schizophrenia and Guha and Nizamie[155] reported clozapine induced seizure.

As in the previous decade, researchers began examining the side-effects of antipsychotics. Ravi *et al*.[156] found that schizophrenics and manics were equally vulnerable to develop neuroleptic-induced dystonia. Kumar and Kumar[157] conducted a similar study in 45 schizophrenic and 45 mood disorder patients. Though dystonia was equally common, pseudoparkinsonism was significantly high in female mood disorder patients, akathisia in middle aged mood disorder patients and tardive dyskinesias in mood disorder patients. Srinivasan and Thomas[158] reported a case of clozapine induced agranulocytosis and treatment by granulocyte-colony stimulating factor.

### Miscellaneous

Chauddury *et al*.[159] found that institutionalized patients were at higher risk of having Hepatitis B infection. Raguram[160] examined the coping strategies employed by schizophrenic patients in a sample of 30 subjects and found that the commonly used techniques were behavioral control, cognitive methods and socialization. The equivalent doses of antipsychotics and their costs were compared by Girish *et al*.[161] and it was found that the antipsychotic drugs were affordable and comparable to drug treatment costs of physical illnesses.

### Comments

Epidemiological work in the 90s established incidence and prevalence rates for schizophrenia in our country, which has been a very significant contribution of this era and has impacted health policies and planning of delivery of mental health services. Biological research continued its directionless replication-oriented path without much impact. Phenomenological research continued in a sound way and had varied topics from clinical descriptions to insight and the like. Course and outcome research consolidated the gains of 80s and attracted national and international attention. Clinical psychological assessment research took a back seat though research in psychosocial and rehabilitation aspects flourished. Introduction of “second generation antipsychotics” ushered in a wave of drug trials that is still pulsating. Clozapine and “treatment resistant schizophrenia” were studied more energetically and with better research designs.

### 2000s

#### Biological research

Like in the previous decade, researchers across the country continued to perform research on various metabolites and hormones. Anand *et al*.[162] measured cerebrospinal fluid levels of dopamine, serotonin and their metabolites and attempted to elucidate their relation to various psychopathological dimensions. Serum prolactin levels, when measured in drug naïve patients, were raised but did not show any relation to severity of psychopathology or prognosis.[163] Dadheech *et al*.[164] studied the levels of antioxidant enzymes in 50 subjects and compared them to normal controls and found deficits in schizophrenic subjects. Singh *et al*.[165] compared oxidative stress and interrelationship of important antioxidants in haloperidol and olanzapine treated patients with schizophrenia. Mishra *et al*.[166] studied neuropsychological profile in schizophrenic patients and suggested that a possibility of combined cerebral dysfunction, more towards left hemispheric dysfunction.

Using neuroimaging and functional imaging, Raju *et al*.[167] carried out magnetic resonance imaging in schizophrenia patients to measure prefrontal lobe volumes in patients with and without frontal dysfunction as measured by the Lubria-Nebraska Neuropsychological Battery and reported that frontal dysfunction cases had minor frontal structural deficits. Padmavati[168] studied differences in cerebral morphology in three groups-untreated schizophrenia patients with dyskinesia, without dyskinesia and normal subjects. Venkatasubramanian *et al*.[169] studied caudate volumes and Venkatasubramanian[170] examined gray matter volumes in patients with schizophrenia using MRI. Seethalakshmi *et al*.[171] carried out functional imaging using FDG-PET in 18 male patients and found that positive syndrome was associated with significantly increased glucose metabolism in medial temporal regions, basal ganglia and left thalamic regions and that chronicity and severity did not influence cerebral glucose metabolism.

Malhotra *et al*.[172] using SPECT in childhood onset schizophrenia found perfusion anomaly in the left temporal, frontal and parietal areas. John *et al*.[173] studied the correlation between psychopathology and EEG alpha coherence in drug naïve recent onset schizophrenia patients and suggested a possible differential pattern in the extent of brain involvement across psychopathological dimensions.

In children of schizophrenia patients, neurobehavioral functioning, social behavior, cognitive function, attention and intelligence were assessed by Shah *et al*.;[174] with more behavioral problems, poor attention and disordered thinking.

Singh *et al*.[175] investigated the incidence of Human Leucocyte Antigen (HLA) Class 1 antigens to understand the role of HLA genes in schizophrenia and found significant increase for HLAA*03 and significant decrease in HLAA*25, A*31 and A*51.

### Phenomenology

Tharyan and Sarvanan[176] studied the relation between insight and psychopathology and found that the severity of psychopathology correlated significantly with dimensional measures of awareness of the abnormal experiences. Armstrongh *et al*.[177] found no relation between insight and psychopathology and severity of illness. Raj and Raguram[178] found that the prevalence of neurotic symptoms in the patient group was high (83.33%) and that there were significant associations between anxiety and certain schizophrenic symptoms. Felt affect in good and poor outcome patients of schizophrenia was compared by Sovani *et al*.[179] and no differences were found. Rajender *et al*.[180] assessed synesthesias and different aspects of body image distortions and their relationship with psychopathology in 70 patients with paranoid schizophrenia and found that synesthesias were positively correlated with disturbances of body concept.

The demographic and clinical correlates of substance abuse in schizophrenia were assessed by Aich *et al*.[181] The substance abusers were predominantly represented by positive syndrome while non-abusers by negative syndrome. Also, the same group[182] found that psychopathology remitted much faster in substance abusing group, but after discharge these patients tended to return back to pre-admission state. In addition, Chakraborty *et al*.[183] found that patients with schizophrenia with substance use tended to have more depressive symptoms in 143 inpatients with ICD-10 diagnosis of schizophrenia.

### Psychology

Sabhesan and Parthasarty[184] studied various dimensions of executive functions in 31 schizophrenia patients and found that they had varying degrees of involvement. On studying neurocognitive dysfunction and REM sleep latency, Das *et al*.[185] found a positive correlation between negative symptoms and neurocognitive deficits and a negative correlation between these parameters and REM latency. On comparing 100 patients with chronic schizophrenia with 100 matched controls on various measures of attention, executive function and memory, Srinivasan *et al*.[186] found that the patient group performed poorly on the tests.

### Psychosocial and rehabilitation

The Quality Of Life (QOL) was assessed by using WHOQOL- BREF in 50 patients by Solanki *et al*.[187], who found that the patients had lowest QOL scores in social relationships. Loganathan and Murthy[188] assessed the experiences of stigma and discrimination in 100 schizophrenia patients and found significant differences between urban and rural respondents. Chandrasekaran *et al*.[189] studied coping in a group of relatives of schizophrenia patients and found that the relatives used resignation, an emotion-focused coping strategy, more commonly than other strategies. Another study in caregivers by Ramohan *et al*.[190] revealed that parents used denial more often and spouses used more of negative distraction strategies for coping. Also, spouses were found to report greater emotional burden. Jayakumar *et al*.[191] found that the caregiver burden was comparable between the caregivers of patients with schizophrenia and obsessive compulsive disorder. Craedo *et al*.[192] assessed the burden and coping of caregivers in relation to the level of functioning in patients with schizophrenia. Fatalism and problem solving were the two most preferred coping mechanisms.

Thomas *et al*.[193] found a significant positive correlation between dysfunction and disruption of family interaction, in a study assessing the extent and pattern of psychosocial dysfunction and family burden in 35 schizophrenia patients. Srivastava *et al*.[194] also studied the perception of burden by caregivers of patients with schizophrenia. Manickam and Chandran[195] assessed the Life Skills Profile (LSP) in schizophrenia patients and found that the rejection responses of key family caregivers were found to be significantly correlated to LSP.

Jagadisha *et al*.[196] found that treatment with antipsychotics was associated with significantly lesser disability. Gandotra *et al*.[197] conducted a study to assess the rehabilitation needs of inpatients and outpatients of schizophrenia and found that negative symptoms significantly correlated with rehabilitation needs in both groups of patients. The impact of vocational rehabilitation in 34 patients with chronic schizophrenia was studied by Kumar[198] who found that there was significant improvement in social functioning, cognitive functioning and psychopathology. Ismail Shihabuddeen and Gopinath[199] assessed the perceived benefits and difficulties of group meetings of caregivers of patients with schizophrenia and bipolar mood disorders. The group meetings led to effective monitoring of the functioning of the individuals, a reduction in the subjective family burden and family distress, a better support system with adequate coping skills and good compliance with treatment. On comparing structured and non-formal family education, Thara *et al*.[200] found that informal educational sessions with periodic across the table re-inforcers may be more effective and practical in the Indian setting.

### Treatment

As in the previous decade, trials with newer antipsychotics continued. Shrivastava and Gopa[201] conducted an open trial comparing risperidone to haloperidol in a sample of 100 patients (50 in each group) over one year. Agarwal and Chadda[202] also found in 44 patients with schizophrenia that once daily risperidone was as efficacious as twice daily risperidone. Another small open trial was done by Avasthi *et al*.[203] comparing olanzapine with haloperidol and found that olanzapine was equally efficacious to haloperidol in improving overall psychopathology and positive symptoms, and had superior efficacy in improving negative symptoms and secondary depressive features. In a double blind trial, Chandra *et al*.[204] compared efficacy of centbutindole and risperidone and found these equally efficacious. A double blind, randomized, prospective trial[205] of 6 weeks’ duration in 46 drug naïve patients comparing risperidone and haloperidol found no significant differences in positive and negative scores; but risperidone was found to have lesser side-effects. An open label, randomized switch over 6 week study[206] of 2 fixed doses (10 mg/15 mg) of aripiprazole was carried out in 136 outpatients with schizophrenia found aripiprazole to be a safe and effective drug. Banerjee *et al*.[207] carried out an open label trial comparing aripiprazole to haloperidol in children and adolescents and found these to be equally efficacious.

Raguraman *et al*.[208] evaluated the effectiveness of clozapine in 22 treatment resistant schizophrenic patients, following them up for 20 months and found that the study group showed better global functioning and decline in suicidal thoughts, negative symptom and general psychopathology scores on PANSS.

With the recognition of obsessive-compulsive symptoms in schizophrenia, Agarwal and Agarwal[209] carried out a small open trial of fluoxetine added to neuroleptic medications to treat obsessive-compulsive symptoms. Vijay Sagar *et al*.[210] evaluated the effects of modafinil add on therapy and found it potentially useful in decreasing weight gain and excessive daytime drowsiness caused by antipsychotics.

Most of the trials were open label trials, with small sample size, with short durations of follow up. Dutta *et al*.[211] observed a multi-drug therapy pattern with a shift towards use of newer atypical antipsychotic agents while assessing psychotropic drug utilization pattern in schizophrenic patients.

Goswami *et al*.[212] carried out a double blind trial of augmentation Electroconvulsive Treatment (ECT) in treatment resistant schizophrenic patients. ECT treated patients improved significantly after the course of six ECTs as compared to those receiving sham-ECT. The efficacy of ECT in lorazepam non-responsive non-affective catatonia was studied in 18 patients by a randomized double blind trial (ECT versus sham ECT).[213] ECT showed superior clinical efficacy and with shorter duration of illness, the response was greater.

Shriharsh *et al*.[214] conducted a study to assess the effects of Cognitive-Behavior Therapy (CBT) on 51 patients suffering from schizophrenia and schizoaffective disorder. They found that CBT sessions resulted in marked improvement in overall adjustment and some improvement in the severity of symptoms; however, the effects were not maintained over a prolonged period.

### Course and outcome

Ponnudurai *et al*.[215] followed up 121 schizophrenia patients, of which 60 were reassessed at 13 years. Seven deaths were recorded and the standardized mortality ratio for all the age groups was 54.2. Philip *et al*.[216] studied the influence of duration of untreated psychosis (DUP) on the short term outcome in schizophrenia patients and found that the DUP was longer in the untreated group.

### Comments

International trends of research in biological aspects were mirrored in research publications of this decade. Neurotransmitter research, neuroimaging, PET scanning, EEG, oxidative stress and antioxidants were investigated and reported with varying degrees of success and impact. Research interest in phenomenology and psychopathology dwindled but “dual diagnosis” i.e. the interface between substance dependence and schizophrenia evoked research fancy of few. Psychometric research had few takers, though enthusiasm in psychosocial and rehabilitation aspects was evidenced by increased number of research publication in these areas during this time frame in question. Drug trials flourished, ECT research declined and other interventions did not attract many researchers.

### Epilogue

From this descriptive annotation it is evident that Indian researchers have meaningfully engaged themselves in schizophrenia research. Phenomenology, clinical and psychosocial variables in schizophrenia are the main strengths of Indian research as reported in our Journal. Of late, coping strategies, stigma and caregiver issues have also emerged as areas of study interest. Significant contribution has been made by Indian researchers in the field of prognosis, course and outcome of schizophrenia that has caught the awareness of the whole world fuelling debate whether or not outcome is genuinely better in the Indian context. Good quality but sporadic biological research in schizophrenia by Indian workers is also documented but cutting edge research in this area still eludes us. Operational research and psychosocial intervention researches are still lagging behind and our talented researchers need to make a move in these areas. Sound research in these areas, complimented by research in health economics of schizophrenia, will impact health policies and delivery of healthcare to this underprivileged, ignored and unfortunate group of patients and their ever so Social caregivers.
